# *Strongyloides stercoralis* combined with concurrent multiple pathogens infections in an immunosuppressed patient: a case report

**DOI:** 10.3389/fmed.2024.1519065

**Published:** 2025-01-08

**Authors:** Jingchun Fang, Huimin Fang, Penghao Guo, Yaqin Peng, Peisong Chen

**Affiliations:** ^1^Department of Laboratory Medicine, The First Affiliated Hospital, Sun Yat-sen University, Guangzhou, China; ^2^Department of Laboratory Medicine, Nansha Division, The First Affiliated Hospital, Sun Yat-sen University, Guangzhou, China

**Keywords:** *Strongyloides stercoralis*, pneumonia, metagenomic next-generation sequencing, co-infection, immunocompromisation

## Abstract

**Background:**

*Strongyloides stercoralis* is an opportunistic pathogenic parasite. Most individuals with normal immune function may not exhibit significant symptoms, and the signs are atypical, which can easily lead to missed diagnoses and delayed treatment. People with underlying diseases and weakened immunity are prone to develop severe conditions after infection with *Strongyloides stercoralis*.

**Case presentation:**

We report an immunocompromised patient in whom the pathogen was initially not detectable using traditional parasitic detection techniques. However, *Strongyloides stercoralis* was identified in both the alveolar lavage fluid and blood through metagenomic next-generation sequencing. Subsequently, *Strongyloides stercoralis* was detected in the alveolar lavage fluid after multiple rounds of testing using traditional microscopic examination techniques. Based on the mNGS results and other examination findings, the patient was diagnosed with *Strongyloides stercoralis* in combination with concurrent multiple pathogens infections. After the combined drug therapy of Meropenem, Vancomycin, and Albendazole, the patient’s condition was gradually brought under control.

**Conclusion:**

This case demonstrates the advantage of integrating traditional detection methods with metagenomics next-generation sequencing technology in the etiological diagnosis of immunocompromised individuals. It is conducive to clarifying the etiological diagnosis of patients and thereby facilitating the timely initiation of corresponding treatments.

## Background

*Strongyloides stercoralis* (*S. stercoralis*) is a soil-transmitted nematode that is endemic to tropical and subtropical regions of the world (1). The lifecycle of *Strongyloides stercoralis* alternates between free-living and parasitic cycles. Under suitable environmental conditions, such as in warm and damp soil, the eggs of the *S. stercoralis* hatch into rhabditiform larvae. After undergoing several molts, these larvae continue to develop into mature adult threadworms. When infective larvae (2) (filariform larvae) enter the human body, they make their way into the circulatory system. They then travel through the right ventricle of the heart to the lungs, where they penetrate the capillaries of the alveolar walls. Then, they move through the bronchial tubes and the pharynx to settle in the small intestine, where they mature and establish themselves. After *S. stercoralis* invades the human body, it can cause strongyloidiasis, and severe cases may even lead to death (3, 4). The diagnosis of *S. stercoralis* infection is difficult because the sensitivity of traditional methods is variable and there is the need to use more techniques such as Baermann concentration, Agar plate culture, Serology and RT-PCR. This paper employs a combination of metagenomic next-generation sequencing (mNGS) and traditional etiological examinations to rapidly and accurately detect a case of *S. stercoralis* co-infection with multiple other pathogens. This approach enables the patient to receive timely and effective treatment and enhances healthcare professionals’ comprehension of co-infections associated with *S. stercoralis*.

## Case presentation

A 75-year-old woman was admitted to the hospital (the First Affiliated Hospital of Sun Yat-sen University, Guangzhou, China) on account of a prolonged cough, expectoration, and recent manifestations of fever and shortness of breath. The patient, a farmer with a generally mediocre health condition, denied any history of exposure to epidemic areas or contaminated water. The patient had a history of COVID-19 infection, hypertension, and nephrotic syndrome. She had been administered irbesartan (150 mg QD), atorvastatin (20 mg QD), methylprednisolone (40 mg QD), and rivaroxaban (10 mg QD) for blood pressure control, lipid regulation, and anticoagulation therapy. Before this admission, she had been on long-term anti-infective therapy at the local hospital, but symptom control was poor. Then the patient presented to the emergency department due to worsening pneumonia, and her condition was complex, requiring airway intubation, mechanical ventilation, and urgent management. In this study, written consent for the publication of detailed information has been obtained from the legal guardians of the patients.

Upon admission, the physical examination revealed the following: body temperature 36.3°C, pulse rate 115 beats per minute, respiratory rate 25 breaths per minute, and blood pressure 95/58 mmHg. The patient was in a state of analgesic sedation, with pale skin and mucous membranes throughout the body, and scattered petechiae were present. The patient had mild edema in the lower extremities. Cardiac and pulmonary auscultation showed no abnormalities, however, coarse breath sounds both dry and wet crackles were heard in both lungs, indicating a lung infection. Abdominal examination showed no significant tenderness, rebound tenderness, or palpable masses, and bowel sounds were normal.

**Figure 1:**
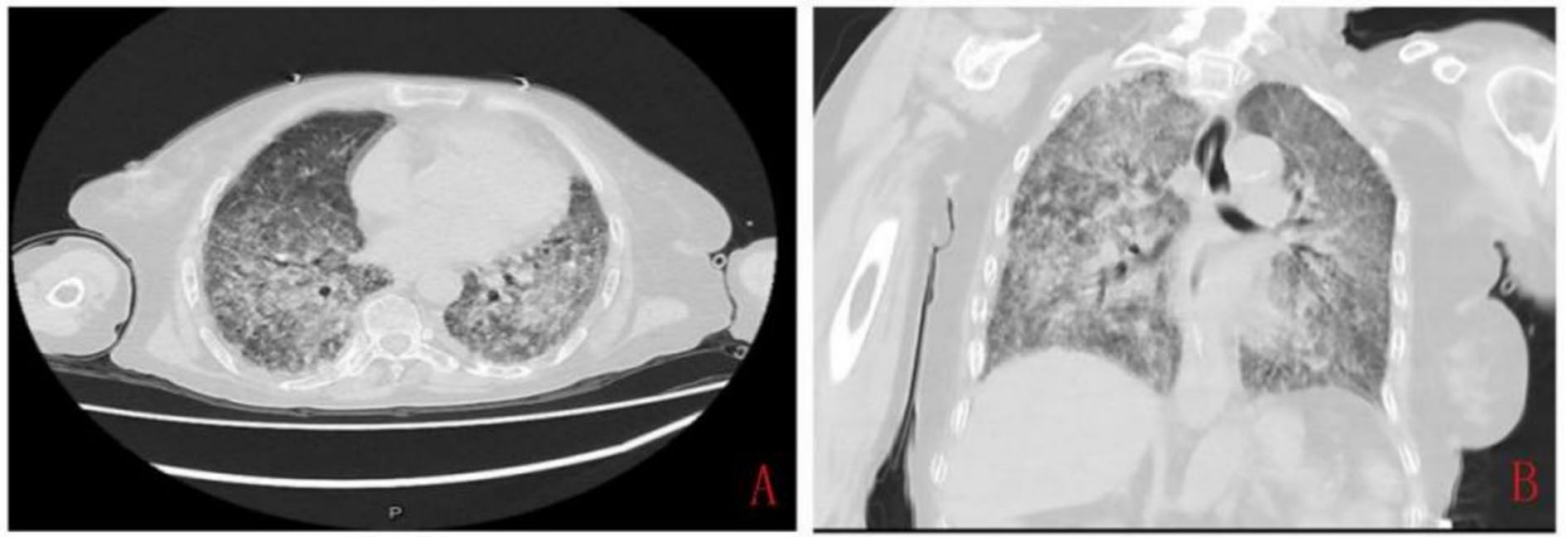
The chest computed tomography imaging at admission showed diffuse inflammation in both lungs. **(A)** Transverse image, showing markedly thickened, disorganized, and blurred pulmonary markings, with some areas presenting a reticular and honeycomb-like appearance. **(B)** Coronal image, showing diffuse patchy areas of increased density in both lungs with indistinct borders.

The Laboratory Examination revealed leukocytosis 4.77 × 10^9^/L, and neutrophils percentage 85.8%, eosinophils percentage 1.2%, increased C-reactive protein (CRP) 51.83 mg/L (normal range 0–10 mg/L), and elevated procalcitonin 2.60 ng/mL (normal range 0–0.05 ng/mL), suggesting a possible infection or inflammation. Coagulation tests indicated prolonged prothrombin time, and gastric juice occult blood test showed a strongly positive result, consistent with gastrointestinal bleeding. Hemoglobin 55 g/L (normal range 115–150 g/L) and serum total protein 53.1 g/L (normal range 64–87 g/L), and serum albumin 31.9 g/L (normal range 35–50 g/L) were decreased. Cellular immunity chip testing showed a decreased CD4+ T cell count of 224 cell/μl (normal range, 500–1,440 cell/μl), CD8+ T cell count of 168 cell/μl (normal range, 238–1,250 cell/μl), and CD3+ T cell count of 420 cell/μl (normal range, 770–2,860 cell/μl), indicating severe cellular immunodeficiency. Chest CT revealed diffuse pulmonary inflammation with a small amount of pleural effusion (Figures 1A,B). Considering that the patient had been on long-term steroid therapy, and Cellular immunity chip testing showed decreased levels of CD3, CD4, and CD8, which indicate cellular immunodeficiency and an increased propensity for infectious diseases, the patient was considered the possibility of pneumonia. Then a combination of Meropenem (1 g iv Q8H), Compound Sulfamethoxazole (0.96 g p.o. Q6H), and Caspofungin (50 mg iv. drip QD) was administered for anti-infection therapy.

On the second day of admission, the patient experienced repeated fevers, with a maximum body temperature of 38.9°C, accompanied by a further increase in CRP (88.71 mg/L) and D-dimer (15.61 mg/L). Subsequently, the blood culture was sent for examination. Furthermore, the patient was tested for Aspergillus antigen, *Cryptococcus neoformans* antigen, rapid detection of *Mycobacterium tuberculosis* and rifampicin resistance gene, acid-fast bacillus smear, *Streptococcus pneumoniae* antigen, Influenza A virus, Influenza B virus, *Mycoplasma pneumoniae*, and *Legionella pneumophila*, all of which were negative. The examination cycles of blood and sputum culture were long, and the results had not been reported yet. Considering the patient was immunocompromised, and her condition was complex, but initial clinical tests failed to detect any pathogens, therefore, mNGS testing of blood and bronchoalveolar lavage fluid (BALF) was sent for the rapid identification of the pathogen. After sample processing and DNA extraction [QIAamp^®^ UCP Pathogen DNA Kit (Qiagen)] for mNGS, libraries were constructed for the DNA samples using a Nextera XT DNA Library Prep Kit (Illumina, San Diego, America), sequencing was performed using Nextseq 550Dx sequencer (Illumina, San Diego, America). The mNGS of BALF identified *Enterococcus faecium* (307,810 reads), *Candida albicans* (296,337 reads), *Candida glabrata* (111,343 reads), *S. stercoralis* (347,139 reads), and *Human Parainfluenza Virus Type 3*, among others (Table 1). Moreover, the blood mNGS detected *S. stercoralis* (15 reads) (Table 2). After examination, the blood culture indicated the presence of *Enterococcus faecium*, and the *in vitro* drug susceptibility test demonstrated sensitivity to vancomycin and linezolid, while the sputum culture disclosed moderate growth of *Candida glabrata.* The other laboratory test results indicated that the patient’s blood contains *Epstein–Barr virus* (EBV) DNA at a level of 1.26 × 10^3^ copies/mL, *Cytomegalovirus* (CMV) DNA at a level of 8.19 × 10^3^ copies/mL, and (1,3)-*β*-D-glucan at a level of 119.58 pg./mL.

**Table 1:** The micro-organisms were detected by mNGS (bronchoalveolar lavage fluid).

Category	Pathogen	Reads	Coverage (%)	Relative abundance (%)
Bacteria	*Enterococcus faecium*	307,810	87.5	87.3
Fungi	*Candida albicans*	296,337	69.2	70.5
	Candida glabrata	111,343	43.8	29.4
Virus	Epstein–Barr virus	262	10.3	58.8
	Human Herpesvirus I	128	5.8	32.4
	Cytomegalovirus	54	1.5	8.8
	Human Parainfluenza Virus Type 3	304,108	100	100
Parasite	Strongyloides stercoralis	347,139	36.2	100
Human microbiota	Hemolytic Staphylococcus	33,869	53.8	9.2
	*Neisseria flavescens*	3,141	31.4	2.8

**Table 2:** The micro-organisms were detected by mNGS (Venous blood).

Category	Pathogen	Reads	Coverage (%)	Relative abundance (%)
Bacteria	–	–	–	–
Fungi	–	–	–	–
Virus	Epstein–Barr virus	3	0.1	3.4
	Cytomegalovirus	115	2.8	94.1
Parasite	Strongyloides stercoralis	15	36.2	100
Human microbiota	–	–	–	–

**Figure 2:**
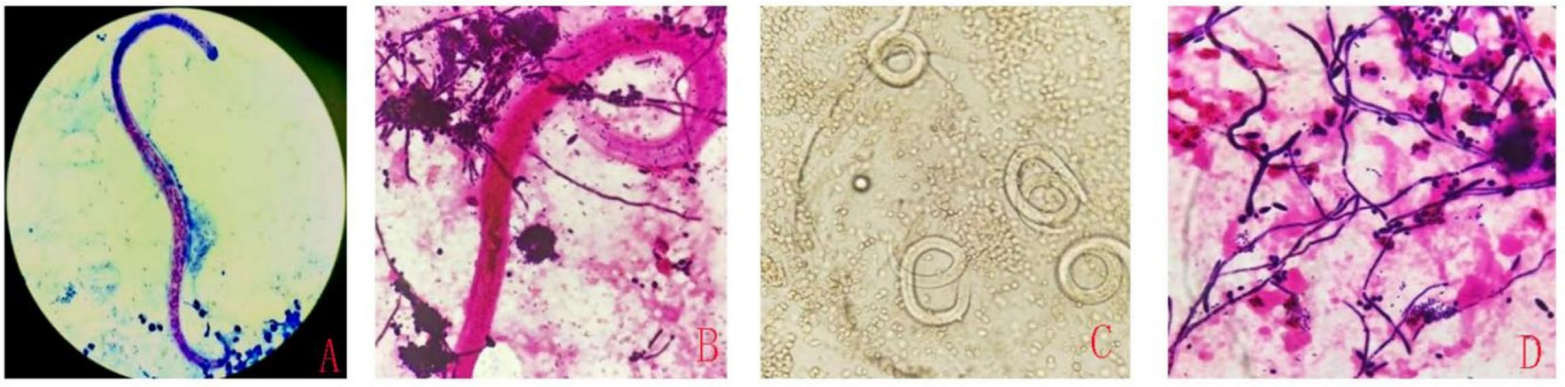
The detection of *S. stercoralis* in alveolar lavage fluid using different smear techniques. Acid-fast staining **(A)**, Gram staining **(B)**, Wet mount observation **(C)**, Gram staining **(D)**.

Based on the mNGS results, a re-examination was conducted on the clinically submitted BALF, and a large quantity of Gram-positive cocci, fungal spores, and pseudohyphae (with observable phagocytosis of white blood cells) were identified. Through repeated smear microscopic examinations, *S. stercoralis* was detected (Figures 2A–D). Nevertheless, the result of the smear test for blood parasites was negative. Regrettably, the patient did not defecate during the hospitalization, so routine stool examination and stool parasite examination could not be performed.

With the mNGS results, the patient was ultimately diagnosed with *S. stercoralis* pneumonia complicated by multiple pathogen infections. BALF NGS and other examination findings suggested multiple bacterial, fungal, viral, and parasitic infections as well as bloodstream infections, prompting an adjustment in the treatment regimen. The patient was administered Meropenem (1 g iv Q8H), Vancomycin (0.5 g iv Q8H), Caspofungin (50 mg iv. drip QD), Oseltamivir (75 mg BID), Ganciclovir (250 mg p.o. Q12H), and Albendazole (0.4 g BID) to enhance antibacterial, antifungal, antiviral and antiparasitic effects.

**Figure 3:**
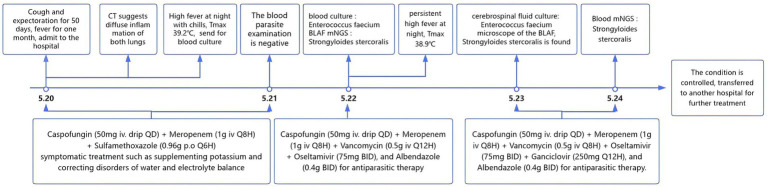
The changes in the whole course of the patient since admission and the process of anti-infection.

With comprehensive treatment, the patient’s vital signs stabilized, infection was controlled. The following figure (Figure 3) shows the changes throughout the entire course of the patient since admission and the process of anti-infection. One week after follow-up, the patient’s infection markers (procalcitonin, 0.673 ng/mL) gradually declined, and the blood culture turned negative, and subsequently, the patient is gradually recovering.

## Discussion

Strongyloidiasis, caused by the parasite *S. stercoralis*, is prevalent worldwide, but it is more common in resource-poor countries with hot and humid climates and poor sanitation conditions (5). This parasite has opportunistic pathogenic characteristics, with the most significant risk factors (5) including HIV infection, *Human T-lymphotropic virus type 1* infection (6), alcoholism, and prolonged soil contact. Generally, most individuals with normal immune function may not exhibit significant symptoms upon infection, while those with underlying health conditions and weakened immunity are more susceptible to developing severe cases (7). The symptoms of infected individuals typically include small localized hemorrhagic spots on the skin, migratory linear or serpiginous urticaria, fever, severe cough with expectoration, coughing, and difficulty breathing (8). Prolonged immunosuppression can escalate to more extensive, hemorrhagic, purpuric lesions, particularly around the umbilicus and limb roots (9). In severe cases of infection, complications such as multi-organ involvement and systemic toxicity may occur, potentially leading to death within a short period. Chest CT scans of those infected may reveal punctate, small patchy, or reticular localized or diffuse inflammatory opacities (10). The patient in this case was elderly and had multiple high-risk factors, including hypertension, nephrotic syndrome, and long-term steroid therapy. The patient had a cough and expectoration for over 50 days, and a chest CT revealed diffuse pulmonary inflammation in both lungs, indicating obvious symptoms of respiratory tract infection. Additionally, the patient presented with pallor of the skin and mucous membranes, scattered petechiae, and localized ecchymosis, suggesting the possibility of infection caused by *S. stercoralis*.

Currently, parasitological techniques are the gold standard for detecting *S. stercoralis* larvae in fecal samples under microscopes (11). However, the sensitivity may be inadequate (12), especially with reduced worm burden, and missed detections were prone to occur. Furthermore, microscopic examination is easily influenced by various factors, such as the specimen quality, the effect of specimen preparation and staining, and the experience of the inspectors. The serology test is also used to diagnose an infection of *S. stercoralis*. Immunodiagnostic tests for strongyloidiasis are indicated when infection is suspected and the organism is not detected by duodenal aspiration, string tests, or by repeated examinations of stool. Most antibody detection tests employ antigens derived from *S. stercoralis* (or from closely-related *S. ratti* or *S. venezuelensis*) filariform larvae, although recombinant antigens such as (e.g., NIE, SsIR) are increasingly being used. Although indirect fluorescent antibody (IFA), indirect hemagglutination (IHA) and antigen-linked fluorescent and magnetic bead tests are are available, enzyme immunoassay (EIA) is recommended because of its greater sensitivity. The filariform antigen-based EIA used at CDC has a sensitivity of 96% and a specificity of 98%. The commercial EIA kits that are currently available have comparable specificity but slightly lower sensitivity. Immunocompromised persons with disseminated strongyloidiasis usually have detectable IgG antibodies despite their immunosuppression, though false negative results can occur.^1^ Hailu et al.’s study indicates that RT-PCR detected the highest number of *S. stercoralis* infections. A combination of RT-PCR with agar plate culture (APC) and/ or Baermann Concentration Test “BCT” better detected *S. stercoralis* from stool samples compared to other combinations or single diagnostic methods. Therefore, RT-PCR and combination of RT-PCR with APC and/or BCT diagnostic methods should be advocated for detection of S. stercoralis infection (13). Molecular techniques can play a confirmatory role in diagnosis, with their ability to circumvent both the low sensitivity of parasitological techniques and the low specificity of immunological techniques (14). The mNGS does not require pre-setting, cultivation, or selectivity. It directly extracts DNA/RNA from clinical samples and completes the detection of pathogens such as bacteria, fungi, viruses, and parasites in one go. This method has diagnostic advantages in populations prone to mixed infections, unexplained critical illnesses and patients with rapid disease progression, particularly those with impaired immune function. In this case, the patient was not initially considered to have a parasitic infection. Due to the deterioration of the condition and unidentified cause of infection, mNGS testing was sent for the rapid identification of the pathogen. Then the *S. stercoralis* was rapidly detected through mNGS. The mNGS provided a direction for the clinical diagnosis and treatment of this patient. Subsequently, based on the mNGS results indicating a significant presence of *Streptococcus constellatus* DNA, multiple and repeated microscopic examinations of the patient’s BALF were conducted. It was through this rigorous and targeted re-examination that we were ultimately able to visualize the parasite under the microscope (Figure 2), confirming the infection caused by *S. stercoralis*. However, the blood parasitic examination remained negative, highlighting the limitations of traditional detection methods in the detection of mixed infections and rare pathogens. A challenge in this case was that the laboratory does not routinely perform smear examinations for BALF cultures, and due to the patient’s condition, it was impossible to obtain feces for routine testing. Through this case, we have recognized the importance of simultaneous microscopic examination for routine cultures.

It is well documented that immunosuppressive agents (e.g., corticosteroids) increase the risk of opportunistic infections (15). On the other hand, several opportunistic infections were reported in COVID-19 patients, including *Candida* spp. (16), *Cytomegalovirus* (CMV) (17), *Herpes simplex virus* (HSV) (18) and *S. stercoralis* (19). In this case, in addition to detecting *S. stercoralis* in the patient’s BALF mNGS results, *Enterococcus, Candida albicans, and Candida glabrata* were also detected. *Candida* spp. are commensal yeasts that are normally found on human skin, in mucosal and intestinal microbiota, and in the mycobiome. and up to 60% of people can be colonized with *Candida* spp. (20) *Candida* spp. can become pathogenic when the equilibrium between commensal organisms is disturbed, and risk factors for *Candida* spp. overgrowth and invasiveness are present. Such risk factors include immunosuppression, the presence of central lines, and exposure to antibiotics (21). As for *Enterococcus*, the most commonly reported infections are intra-abdominal infections, urinary tract infections, bacteremia and endocarditis, pneumonia is rarely described (22). Infections typically present in immunosuppressed patients who have received multiple courses of antibiotics in the past. It is generally believed that *Enterococcus and Candida* have a higher probability of colonization in the respiratory tract, and they generally do not require treatment. However, since the patient was immunocompromised, had previously been infected with COVID-19 and exposed to broad-spectrum antibiotics, the risk of opportunistic infections is relatively high. Based on the microscopic examination of the patient’s sputum, which showed a large number of white blood cells, Gram-positive cocci being engulfed by white blood cells, and Candida hyphae were also visible. Combined with the elevated results of fungal serum (1,3)-β-D-glucan (119.58 pg./mL) and procalcitonin (2.6 ng/mL), empirical treatment targeting both *Enterococcus* and *Candida* is currently being considered.

Studies have indicated that strongyloidiasis complicated by CMV (23) infection often presents with non-specific gastrointestinal symptoms. CMV infection triggers a Th1 type cellular immune response and suppresses the Th2 type cellular immune response associated with *S. stercoralis* infection, which can increase the risk of disseminated strongyloidiasis (8, 24). *S. stercoralis* primarily causes intestinal disease and disrupts the intestinal immune microenvironment, increasing the body’s susceptibility to intestinal bacteria. When both infections occur together, they may complicate the disease course (25). Clinical manifestations in patients with multiple pathogen co-infections are often non-specific, making clinical diagnosis and treatment more challenging (26). Therefore, for patients with compromised immune function (such as the immunosuppressed host with pneumonia in this case) who are prone to various types of opportunistic infections, it is necessary to pay attention to not only common pathogen infections but also to the infections caused by less common pathogens like fungi and parasites (27).

Eosinophils are one of the foremost components of the immune system, which play a prominent role in parasitic infections. Eosinophilia is a common, but not uniform, finding in *S. stercoralis* infection and is thought to be more marked in earlier infections, becoming less pronounced and more variable in chronic cases (28). However, previous reports suggest that patients who have an absence of eosinophilia in the setting of a Strongyloides infection tend to have a poorer prognosis (29). The reason for this observation is unclear, but it may be related to corticosteroid-induced neutrophilia and the fact that corticosteroids can promote the apoptosis of eosinophils (30). The patient’s eosinophil count was normal upon admission, which might be associated with the patient’s disease course, severity of the condition, and the use of glucocorticoids. Following the initiation of the anti-infective therapy, continuous monitoring of the complete blood count over several days disclosed eosinophil levels above the normal range, suggesting a favorable trend in the disease course and the efficacy of the treatment regimen. Through the timely administration of antibacterial, antifungal, antiviral, and antiparasitic treatments, the patient’s condition was controlled.

We are aware that this report has limitations. Firstly, our clinical physicians had ordered routine stool tests, stool parasitic examinations, and stool culture tests early on. Regrettably, the patient did not defecate during the hospitalization, so routine stool examination and stool parasite examination could not be performed. Secondly, the specific cause of the patient’s infection with *S. stercoralis* remains unclear. According to the medical history, we understand that the patient is a farmer, and we can only speculate that the patient might have been exposed to soil contaminated with *S. stercoralis*.

## Conclusion

This case underlines the need to exclude *S. stercoralis* infection particularly in immunocompromised patient with risk factors and highlights the diagnostic power of NGS although it indicates the need not to lose knowledge of traditional methods.

## Data Availability

The datasets presented in this study can be found in online repositories. The names of the repository/repositories and accession number(s) can be found in the article/supplementary material.

## Footnotes

^1^ https://www.cdc.gov/dpdx/strongyloidiasis/index.html
